# European multicenter study on antimicrobial resistance in bacteria isolated from companion animal urinary tract infections

**DOI:** 10.1186/s12917-016-0840-3

**Published:** 2016-09-22

**Authors:** Cátia Marques, Luís Telo Gama, Adriana Belas, Karin Bergström, Stéphanie Beurlet, Alexandra Briend-Marchal, Els M. Broens, Marta Costa, Delphine Criel, Peter Damborg, Marloes A. M. van Dijk, Astrid M. van Dongen, Roswitha Dorsch, Carmen Martin Espada, Bernhard Gerber, Maria Kritsepi-Konstantinou, Igor Loncaric, Domenico Mion, Dusan Misic, Rebeca Movilla, Gudrun Overesch, Vincent Perreten, Xavier Roura, Joachim Steenbergen, Dorina Timofte, Georg Wolf, Renato Giulio Zanoni, Sarah Schmitt, Luca Guardabassi, Constança Pomba

**Affiliations:** 1 Faculdade de Medicina Veterinária, Centro de Investigação Interdisciplinar em Sanidade Animal (CIISA), Lisboa, Portugal; 2 The National Veterinary Institute (SVA), Uppsala, Sweden; 3 Laboratoire Vebiotel, Arcueil, France; 4 Department of Infectious Diseases and Immunology, Utrecht University, Utrecht, The Netherlands; 5 Department of Clinical Veterinary Sciences, Faculty of Medical and Veterinary Sciences, University of Bristol, Langford, UK; 6 AML-Medvet laboratory, Antwerp, Belgium; 7 Department of Veterinary Disease Biology, Faculty of Health and Medical Sciences, University of Copenhagen, Frederiksberg, Denmark; 8 Department of Clinical Sciences of Companion Animals, University of Utrecht, Utrecht, The Netherlands; 9 Clinic of Small Animal Medicine, LMU Munich, Munich, Germany; 10 Hospital Clínico Veterinario Complutense, Universidad Complutense, Servicio de Microbiologia y Parasitologia, Madrid, Spain; 11 Vetsuisse Faculty, University of Zurich, Clinic for Small Animal Internal Medicine, Zurich, Switzerland; 12 Department of Clinical Studies, Companion Animal Clinic, Faculty of Health Sciences, School of Veterinary Medicine, Aristotle University of Thessaloniki, Thessaloniki, Greece; 13 Institute of Microbiology, University of Veterinary Medicine, Vienna, Austria; 14 Department of Veterinary Medical Science, University of Bologna, Bologna, Italy; 15 Department of microbiology, Faculty of Veterinary Medicine, University of Belgrade, Belgrade, Serbia; 16 Hospital Clínic Veterinari, Universitat Autònoma de Barcelona, Bellaterra, Spain; 17 Institute of Veterinary Bacteriology, Vetsuisse Faculty, University of Bern, Bern, Switzerland; 18 School of Veterinary Science, University of Liverpool, Cheshire, UK; 19 Faculty of Veterinary Medicine, University of Agronomical Sciences Veterinary Medicine, Lasi, Romania; 20 Institute of Infection and Global Health, University of Liverpool, Liverpool, UK; 21 Institute for Infectious Diseases and Zoonoses, LMU Munich, Munich, Germany; 22 Institute of Veterinary Bacteriology, Vetsuisse Faculty, University of Zurich, Zurich, Switzerland; 23 Department of Biomedical Sciences, Ross University School of Veterinary Medicine, Basseterre, Saint Kitts and Nevis

**Keywords:** Antimicrobial resistance, Temporal trends, MRSA, MRSP, Dog, Cat

## Abstract

**Background:**

There is a growing concern regarding the increase of antimicrobial resistant bacteria in companion animals. Yet, there are no studies comparing the resistance levels of these organisms in European countries. The aim of this study was to investigate geographical and temporal trends of antimicrobial resistant bacteria causing urinary tract infection (UTI) in companion animals in Europe. The antimicrobial susceptibility of 22 256 bacteria isolated from dogs and cats with UTI was determined. Samples were collected between 2008 and 2013 from 16 laboratories of 14 European countries. The prevalence of antimicrobial resistance of the most common bacteria was determined for each country individually in the years 2012–2013 and temporal trends of bacteria resistance were established by logistic regression.

**Results:**

The aetiology of uropathogenic bacteria differed between dogs and cats. For all bacterial species, Southern countries generally presented higher levels of antimicrobial resistance compared to Northern countries. Multidrug-resistant *Escherichia coli* were found to be more prevalent in Southern countries. During the study period, the level of fluoroquinolone-resistant *E. coli* isolated in Belgium, Denmark, France and the Netherlands decreased significantly. A temporal increase in resistance to amoxicillin-clavulanate and gentamicin was observed among *E. coli* isolates from the Netherlands and Switzerland, respectively. Other country-specific temporal increases were observed for fluoroquinolone-resistant *Proteus* spp. isolated from companion animals from Belgium.

**Conclusions:**

This work brings new insights into the current status of antimicrobial resistance in bacteria isolated from companion animals with UTI in Europe and reinforces the need for strategies aiming to reduce resistance.

## Background

Bacterial urinary tract infections (UTI) are frequently diagnosed in dogs and are considered rare in cats [1, 2]. Lately, increased frequencies of UTI in cats have been reported in some European countries [3–5] in particularly when concurrent diseases are present [6].

*Escherichia coli* is the most frequent isolated bacteria causing UTI in dogs and cats. Several studies show frequencies greater than 30 % [7–9]. Other commonly isolated bacteria genera include *Staphylococcus* spp., *Enterococcus* spp., *Proteus* spp. and *Klebsiella* spp. [7–10].

Previous studies in the United Kingdom and in Missouri-Columbia (USA) analysing the temporal trends of antimicrobial resistance in small collections of bacterial isolates from companion animal infections point to a significant increase in antimicrobial resistance [11, 12]. Furthermore, the emergence of multidrug-resistant bacteria (isolates resistant to three or more antimicrobial categories) in companion animals is an increasing concern [11, 13–15]. This creates new therapeutic challenges in veterinary medicine and is also a public health issue, since these pathogens may be zoonotic [16] and companion animals may play a role in the spread of resistant bacteria due to their close contact to humans [14, 17].

Antimicrobial resistance may vary according to the geographic location [9, 18]. Data on antimicrobial resistance in bacteria isolated from companion animals with UTI in Europe are not easily comparable due to differences in study design, such as variations in host species, inclusion criteria and/or time period. Thus, it is difficult to get a European overview of antimicrobial resistance as seen in human medicine surveillance programmes such as the European Antimicrobial Resistance Surveillance Network [18].

Antimicrobial therapy in UTI should ideally rely on susceptibility testing of the isolated bacteria [19]. Yet, antimicrobials are frequently administered empirically based on the presence of compatible clinical signs, urine cytological findings and in the absence of urine culture and are required to alleviate UTI symptoms while waiting for antimicrobial susceptibility testing results [19]. Besides the pharmacokinetic-pharmacodynamic properties, the empiric antimicrobial selection should consider the most likely causative agent as well as its regional susceptibility patterns [8]. Moreover, according to the World Organisation for Animal Health [20], veterinarians should adopt strategies aimed at the reduction of antimicrobial resistance. Therefore, current information on the aetiology and antimicrobial resistance focused on UTI is of crucial importance.

Under the umbrella of the European Society of Veterinary Nephrology and Urology, a multicenter retrospective study was launched in November 2013 with the goal of getting antimicrobial resistance data on bacteria isolated from companion animal with UTI across Europe. A Urinary Tract Infection Resistance – Veterinary Network (UTIR-VNet) was constituted with this purpose in mind. Partial results were presented at the annual Society meeting included in the 25^th^ congress of the European College of Veterinary Internal Medicine, 4–6 September 2014, Mainz, Germany. The aim of this study was to determine the frequency of uropathogens in dogs and cats with urinary tract infection in Europe and to characterise the frequency and temporal trends of antimicrobial resistance over a period of six years. We hereby present a complete report and discussion of this study.

## Methods

### Participating countries

**Fig. 1:**
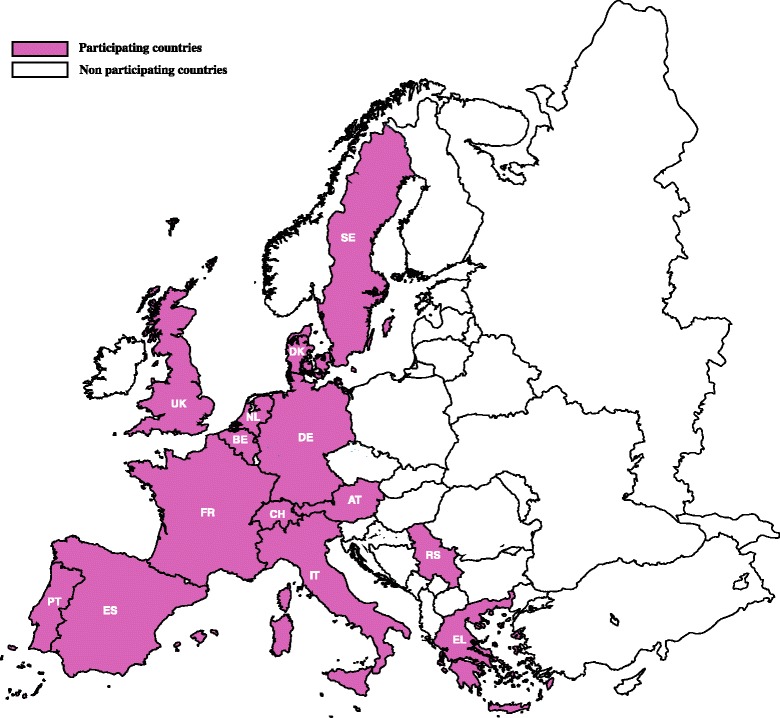
Participating countries in the Urinary tract infection antimicrobial resistance veterinary network – UTIR-VNet. Countries: AT- Austria; BE- Belgium; DK- Denmark; FR- France; DE- Germany; EL- Greece; IT- Italy; NL- the Netherlands; PT- Portugal; RS- Serbia; ES- Spain; SE- Sweden; CH- Switzerland; UK- United Kingdom

Between January and September 2014, 16 veterinary microbiology laboratories from 14 European countries (Austria, Belgium, Denmark, France, Germany, Greece, Italy, the Netherlands, Portugal, Serbia, Spain, Sweden, Switzerland, United Kingdom), were invited to participate in this study (Fig. 1). Laboratories were requested to send available retrospective data on animal species, age and gender, bacterial identification and antimicrobial susceptibility testing conducted in bacteria obtained from dogs and cats with UTI between 2008 and 2013. Samples were obtained with owners consent as part of the routine care of canine and feline UTI.

### Bacterial Isolates

The bacteria identification varied between laboratories. Most laboratories used standard phenotypic tests, including API, while others used techniques such as PCR and MALDI-TOF. This discrepancy was particularly evident for staphylococci, which were classified to either the species or genus level depending on the laboratory.

### Susceptibility testing

The following antimicrobials were included: amoxicillin-clavulanate (AMC), ampicillin (AMP), cefotaxime (CTX), cefovecin (CVN), cefoxitin (FOX), ceftazidime (CAZ), cefpodoxime (CPD), ceftiofur (EFT), ciprofloxacin (CIP), enrofloxacin (ENR), gentamicin (CN), marbofloxacin (MAR), oxacillin (OX), penicillin (P) and trimethoprim/sulfamethoxazole (SXT).

The retrospective nature of the study forced us to include two in vitro antimicrobial susceptibility testing methods. Laboratories from Austria, France, Germany, Greece, Italy, the Netherlands, Portugal, Serbia, Spain, United Kingdom, used standard disc diffusion method according to Clinical Laboratory Standards Institute (CLSI) guidelines [21], whereas Sweden (VetMIC, SVA, Uppsala, Sweeden), Denmark and United Kingdom (COMPAN1F Sensititre panels, Thermo Fisher), Switzerland and Belgium (VITEK 2, BioMérieux) used broth microdilution method.

Human CLSI breakpoints [22] were used for interpretation of minimal inhibitory concentration and disk diffusion results for CAZ (30 μg), CTX (30 μg), and CIP (5 μg), whereas veterinary CLSI breakpoints [23] were used for AMC (30 μg), AMP (30 μg), CN (10 μg), CPD (10 μg), ENR (5 μg), FOX (30 μg), MAR (5 μg), OX (1 μg), P (10U), and SXT (25 μg). Clinical breakpoints from the Societé Française de Microbiology [24] were used for EFT (30 μg). Results for CVN (30 μg) were interpreted according to the manufacturer guidelines. As seen in the human European Antimicrobial Resistance Surveillance Network (EARS-Net) report [18], when data on minimal inhibitory concentrations or inhibition zone diameter were not available, the laboratories’ own interpretations (susceptible, intermediate and resistant) were accepted. This was the case for Spain, Serbia, and Germany that used contemporary CLSI guidelines, for the United Kingdom that used the breakpoints from the British Society of Antimicrobial Chemotherapy [25] and for the Netherlands and Switzerland (2008–2010) that used breakpoints recommended by the Dutch Committee on Guidelines for Susceptibility testing [26].

### Data analysis and statistical methods

Statistical analysis was performed using the SAS statistical software package for Windows, version 9.3, (SAS Institute Inc, Cary, North Carolina, USA).

The Fisher exact test was used to compare pathogen frequencies by species or gender of the host, by simple/multiple infection and by country. An alpha value of 0.05 was used.

Isolates were considered fully resistant when found to be resistant according to the clinical breakpoint applied. An isolate was considered susceptible when found to be susceptible or intermediate according to the clinical breakpoint applied. The antimicrobials included in this study are known to be highly concentrated in the urine so their report as susceptible may be appropriated for isolates categorized as intermediate [19].

Regarding third generation cephalosporins (3GC), laboratories tested different 3GC resistance surrogates. Therefore, to evaluate the antimicrobial resistance to 3GC, an isolate was considered as 3GC resistant when it was resistant to at least one of the five 3GC tested (CTX, CAZ, CVN, EFT or CPD). The same rational was applied to evaluate resistance to fluoroquinolones (FLU), namely using ENR, CIP or MAR as a marker of resistance. Methicillin-resistance in staphylococci was determined according to CLSI guidelines [23] using cefoxitin or oxacillin to evaluate resistance depending on the bacterial species considered. Yet, Germany and Spain did not send data on methicillin-resistance. France did not test staphylococci against oxacillin and thus did not report methicillin-resistance regarding *Staphylococcus pseudintermedius* (MRSP). The Netherlands did not have data on staphylococci susceptibility to OX or FOX but instead reported data on the detection of the *mec*A gene by PCR. The frequency of methicillin-resistance did not include staphylococci only identified to the genus level.

Enterobacteriaceae were considered multidrug-resistant (MDR) when fully resistant to three or more categories of antimicrobials, namely AMC, 3GC, SXT, CN and/or FLU. Unlike the MDR definition proposed by other authors [27], intermediate isolates from this study were considered as susceptible. This difference was applied because we are considering drugs that can be highly concentrated in urine. Furthermore, this approach will reduce any overestimation of MDR frequency due to the use of different breakpoint guidelines. Full-susceptibility (FullS) was defined as an isolate being susceptible for all the above-mentioned categories of antimicrobials. Since Belgium had no data available on 3GC and the Netherlands had little data on CN, MDR and FullS percentages do not include resistance to 3GC for Belgium and resistance to CN for the Netherlands.

As a rule, statistical analysis was only done when at least ten isolates for a specific organism-antimicrobial agent combination were reported for a given country. All frequencies are presented with a confidence interval of 95 % (95 % CI).

Maps of European resistance distribution were drawn considering the percentage of fully resistant isolates to the considered antimicrobial agent, over the years 2012–2013. A scale of colours was applied composed of six resistance intervals after the example of EARS-Net surveillance program reports [18].

Statistical analysis of temporal trends of antimicrobial resistance for a specific organism-antimicrobial agent combination were determined within each country. Temporal trends were only determined for countries reporting data on at least three consecutive years and ten isolates per year. A SAS LOGISTIC regression, with the year as a continuous variable and an alpha value of 0.05 was conducted. Temporal trends of resistance were mainly determined for *E. coli* since this was the most represented bacterial species. Yet, temporal trends of AMC, FLU and SXT in *Proteus* spp. were also determined for Belgium, France, the Netherlands and Sweden.

## Results

Overall, data on 22,256 uropathogenic bacteria were obtained from 15,097 dog and 5963 cat positive urine cultures. Table 1 summarises the numbers of bacterial isolates obtained by year and country.

**Table 1:** Total number of isolated bacteria by year and country

Country^a^	Year						
	2008	2009	2010	2011	2012	2013	Total
AT	-	-	-	-	144	185	329
BE	-	-	547	578	623	739	2487
DK	29	30	53	116	153	205	587
FR	-	-	620	733	780	995	3128
DE	-	64	93	140	161	146	604
EL	24	29	13	11	32	43	152
IT	-	36	29	36	77	65	243
NL	480	867	958	1132	1195	1307	5939
PT	77	54	57	34	32	45	299
RS	17	19	10	2	3	3	54
ES	14	23	27	40	47	79	230
SE	730	924	1071	1202	1355	1647	6929
CH	109	120	112	125	114	174	754
UK	31	44	81	117	126	122	521
Total	1511	2210	3671	4267	4842	5755	22256

^a^AT, Austria; BE, Belgium; DK, Denmark; FR, France; DE, Germany; EL, Greece; IT, Italy; NL, the Netherlands; PT, Portugal; RS, Serbia; ES, Spain; SE, Sweden; CH, Switzerland; UK, United Kingdom

Considering the records containing information about the age, dogs (*n* = 4425) and cats (*n* = 1514) had similar mean ages, namely 8.77 years (SD ± 4.04, 9.00 median, 6.00 IQR, range 0.1–20) and 8.82 years (SD ± 5.03, 8.50 median, 8.1 IQR, range 0.2–22) respectively. Gender was only specified in 3885 records where 61.41 % (95 % CI 59.69–63.12 %, *n* = 1900/3094) of dogs and 48.29 % (95 % CI 44.81–51.78 %, *n* = 382/791) of cats were females.

Among all urine cultures, 94.64 % (95 % CI 94.33–94.94 %, *n* = 19932/21060) resulted in the growth of bacterial pure cultures, with no significant difference between cats and dogs (*P* = 0.1856). Both in dogs and cats, *E. coli* was the most frequently identified bacteria and accounted for 59.45 % (95 % CI 58.80–60.09 %, *n* = 13231/22256) of all isolates. The frequency of the remaining bacterial species differed significantly between dogs and cats (Table 2). *Enterococcus* spp. and *Staphylococcus* spp. frequencies were higher in cats, whereas *Proteus* spp. and *Klebsiella* spp. were more prevalent in dogs.

**Table 2:** Uropathogenic bacteria aetiology, single versus mixed infections and cat versus dog as host species

Organism	Overall		Single organism		Mixed infections			Dogs		Cats		
	*n*	% (95 % CI)^a^	*n*	% (95 % CI)^a^	*n*	% (95 % CI)^a^	*P*	*n*	% (95 % CI)^a^	*n*	% (95 % CI)^a^	*P*
*Enterobacter* spp.	308	1.38 (1.23–1.54)	244	1.22 (1.07–1.38)	64	2.75 (2.09–3.42)	**<0.0001**	194	1.21 (1.04–1.38)	114	1.81 (1.48–2.14)	**0.0008**
*Enterococcus* spp.	1506	6.77 (6.44–7.10)	1129	5.66 (5.34–5.99)	377	16.22 (14.72–17.72)	**<0.0001**	745	4.66 (4.34–4.99)	761	12.11 (11.31–12.92)	**<0.0001**
*Escherichia coli*	13231	59.45 (58.80–60.09)	12417	62.30 (61.62–62.97)	814	35.03 (33.09-36-97)	**<0.0001**	9506	59.51 (58.75–60.27)	3725	59.30 (58.08–60.51)	0.7832
*Klebsiella* spp.	478	2.15 (1.96–2.34)	400	2.01 (1.81–2.20)	78	3.36 (2.62–4.09)	**<0.0001**	385	2.41 (2.17–2.65)	93	1.48 (1.18–1.78)	**<0.0001**
*Proteus* spp.	1992	8.95 (8.58–9.33)	1770	8.88 (8.49–9.28)	222	9.55 (8.36–10.75)	0.2824	1869	11.70 (1.20–1.22)	123	1.96 (1.62–2.30)	**<0.0001**
*Pseudomonas* spp.	389	1.75 (1.58–1.92)	315	1.58 (1.41–1.75)	74	3.18 (2.47–3.90)	**<0.0001**	293	1.83 (1.63–2.04)	96	1.53 (1.22–1.83)	0.1249
*Staphylococcus* spp.	2893	13.00 (12.56–13.44)	2519	12.64 (12.18–13.10)	374	16.09 (14.60–17.59)	**<0.0001**	1836	11.49 (11.00–11.99)	1057	16.83 (15.90–17.75)	**<0.0001**
*Streptococcus* spp.	802	3.60 (3.36–3.85)	586	2.94 (2.71–3.17)	216	9.29 (8.11–10.47)	**<0.0001**	675	4.23 (3.91–4.54)	127	2.02 (1.67–2.37)	**<0.0001**
Other	657	2.95 (2.73–3.17)	552	2.77 (2.54–3.00)	105	4.52 (3.67–5.36)	-	471	2.95 (2.69–3.21)	186	2.96 (2.54–3.38)	-

^a^95 % CI, 95 % Confidence interval

*n* – Total number of isolates

*P*- P value obtained by Fisher exact test when comparing single versus mixed infections and cat versus dog as host. Statistically significant values are highlighted in bold

**Fig. 2:**
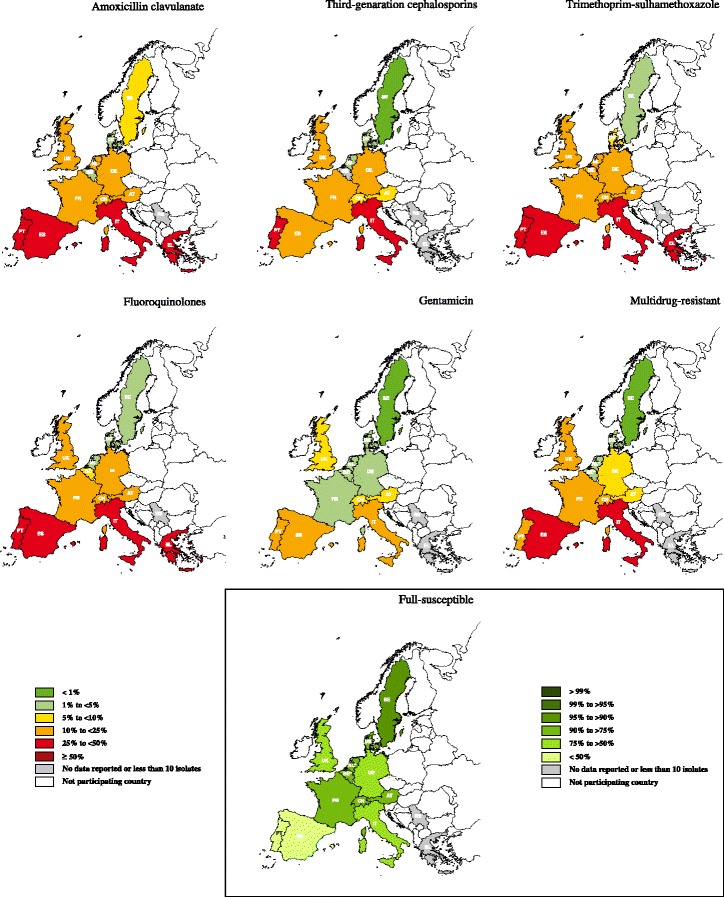
Percentage (%) of *Escherichia coli* antimicrobial resistance by antimicrobial and country in the years 2012–2013. Countries: AT- Austria; BE- Belgium; DK- Denmark; FR- France; DE- Germany; EL- Greece; IT- Italy; NL- the Netherlands; PT- Portugal; RS- Serbia; ES- Spain; SE- Sweden; CH- Switzerland; UK- United Kingdom. Multidrug-resistance considering combined resistance to three or more of the following antimicrobial categories: AMC, 3GC, FLU, CN and SXT. Full-susceptibility (FullS) was defined as an isolate being susceptible for all the above-mentioned categories of antimicrobials. Regarding multidrug-resistance and full-susceptibility frequencies, countries marked by asterisk: 3GC was not included for Belgium and CN for the Netherlands. Thus, these frequencies may be underestimated when compared with the remaining countries

**Fig. 3:**
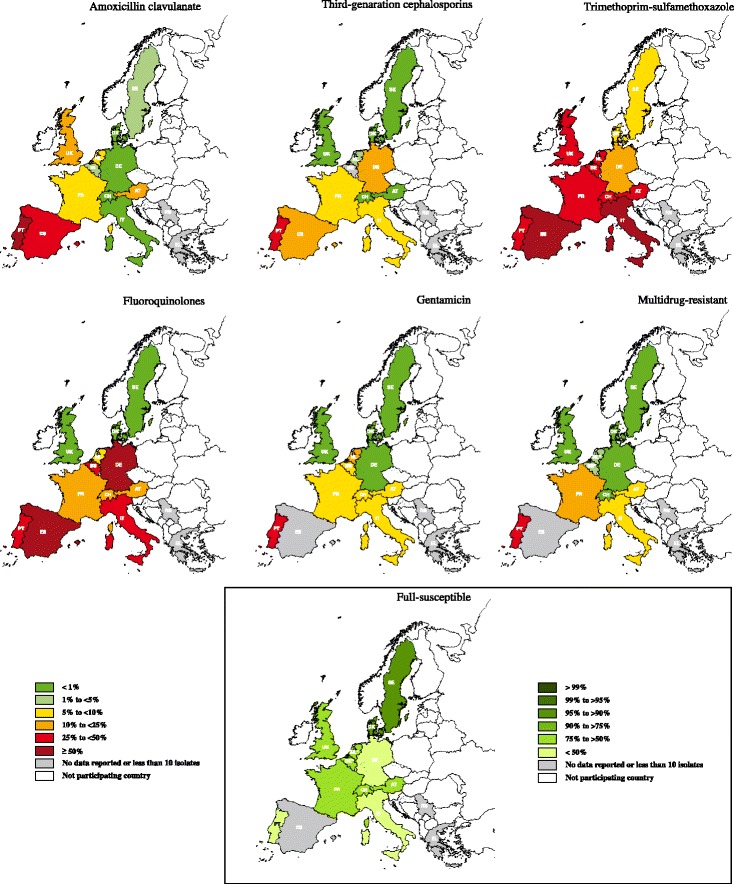
Percentage (%) of *Proteus* spp. antimicrobial resistance by antimicrobial and country in the years 2012–2013. Countries: AT- Austria; BE- Belgium; DK- Denmark; FR- France; DE- Germany; EL- Greece; IT- Italy; NL- the Netherlands; PT- Portugal; RS- Serbia; ES- Spain; SE- Sweden; CH- Switzerland; UK- United Kingdom. Multidrug resistance considering combined resistance to three or more of the following antimicrobial categories: AMC, 3GC, FLU, CN and SXT. Full-susceptibility was defined as an isolate being susceptible for all the above-mentioned categories of antimicrobials. Regarding multidrug-resistance and full-susceptibility frequencies, countries marked by asterisk: 3GC was not included for Belgium and CN for the Netherlands. Thus, these frequencies may be underestimated when compared with the remaining countries

Considering the years 2012–2013, the major differences in *E. coli* (Table 3, Fig. 2) and *Proteus* spp. (Table 4, Fig. 3) antimicrobial resistance frequencies were seen between Northern (Denmark and Sweden) and Southern (Italy, Greece, Portugal and Spain) countries.

**Table 3:** Percentage of resistance in *Escherichia coli* by antimicrobial and country in 2012–2013

Country^a^	AMC		3GC		FLU		CN		SXT		Combined resistance		
	*n*	% R (95 % CI)^b^ [Stat. Dif.]^c^	*n*	% R (95 % CI)^b^ [Stat. Dif.]^c^	*n*	% R (95 % CI)^b^ [Stat. Dif.]^c^	*n*	% R (95 % CI)^b^ [Stat. Dif.]^c^	*n*	% R (95 % CI)^b^ [Stat. Dif.]^c^	*n*	% MDR (95 % CI)^b^ [Stat. Dif.]^c^	% FullS (95 % CI)^b^ [Stat. Dif.]^c^
AT	142	14.08 (8.36–19.81) [a,b]	142	5.63 (1.84–9.43) [a, b]	142	11.97 (6.63–17.31) [a]	142	5.63 (1.84–9.43) [a, b]	142	14.08 (8.36–19.81) [a, b]	142	8.45 (3.88–13.03) [a]	78.87 (72.16–85.59) [a]
BE	840	4.29 (2.92–5.66) [c]	0	- -	769	6.63 (4.87–8.39) [b]	840	1.67 (0.80–2.53) [c]	839	10.37 (8.31–12.43) [a]	769^d^	1.43^d^ (0.59–2.27) -	85.05^d^ (82.52–87.57) -
DK	206	2.88 (0.61–5.16) [c]	208	4.33 (1.41–7.09) [a, c]	208	2.88 (0.61–5.16) [c]	208	1.92 (0.06–3.79) [a, c]	208	8.17 (4.45–11.90) [a, c]	208	2.88 (0.61–5.16) [b]	88.94 (84.68–93.20) [b, c]
FR	954	12.79 (10.67–15.91) [a, d]	933	10.83 (8.83–12.82) [b]	948	12.76 (10.64–14.89) [a]	951	3.36 (2.22–4.51) [a, d]	959	16.27 (13.93–18.60) [b, d]	909	11.00 (8.97–13.04) [a, c]	77.23 (74.50–79.95) [a]
DE	153	11.76 (6.66–16.87) [a, d]	152	11.84 (6.71–16.98) [b, d]	153	16.34 (10.48–22.20) [a, d]	153	1.96 (0.00–4.16) [a, c, d]	153	17.65 (11.61–23.69) [b, d, e]	152	8.55 (4.11–13.00) [a]	67.76 (60.33–75.19) [d]
EL	31	25.81 (10.40–41.21) [b, d, e, f]	9	7R/2S -	30	30.00 (13.60–46.40) [d, e]	0	- -	26	34.62 (16.33–52.90) [e, f]	0	- -	- -
IT	69	26.09 (15.73–36.45) [e, f]	69	24.64 (14.47–34.80) [e]	69	31.88 (20.89–42.88) [e]	69	14.49 (6.19–22.80) [e]	69	28.99 (18.28–39.69) [e, f]	69	28.99 (18.28–39.69) [d]	63.77 (52.43–75.11) [d, e, f]
NL	1461	10.81 (9.22–12.41) [a]	1380	3.77 (2.76–4.77) [a, c]	1457	4.94 (3.83–6.05) [b, c]	81	3.70 (0.00–7.82) [a, c, f]	1459	10.21 (8.66–11.77) [a]	1380^d^	2.25^d^ (1.46–3.03) -	81.30^d^ (79.25-83.36) -
PT	27	48.15 (29.30-66.99) [e]	32	31.25 (15.19-47.31) [e]	31	29.03 (13.05-45.01) [d, e]	30	10.00 (0.00-20.74) [b, d, e, f]	31	32.26 (15.80-48.71) [e, f]	25	24.00 (7.26-40.74) [c, d, e]	32.00 (13.71–50.29) [g, h]
RS	3	2R/1S -	2	1R/1S -	3	0R/3S -	3	0R/3S -	3	1R/2S -	2	1MDR -	1Full-S -
ES	60	31.67 (19.90–43.44) [e, f]	52	21.15 (10.05–32.25) [d, e]	61	29.51 (18.06–40.95) [e]	46	15.22 (4.84–25.60) [b, e]	60	26.67 (15.48–37.86) [e, f]	37	29.73 (15.00–44.46) [d, e]	43.24 (27.28–59.21) [e, g]
SE	2091	6.98 (5.89–8.07) [g]	2082	0 [f]	2091	1.05 (0.61–1.49) [f]	2091	0.19 (0.00–0.38) [g]	2091	4.97 (4.04–5.91) [c]	2082	0.24 (0.03–0.45) [f]	90.2 (88.92–91.48) [b, h]
CH	133	10.53 (5.31–15.74) [a, g]	133	8.27 (3.59–12.95) [b, c]	132	13.64 (7.78–19.49) [a, d]	132	6.82 (2.52–11.12) [b, d, e, f]	131	13.74 (7.85–19.64) [a, d, g]	130	10.00 (4.84–15.16) [a, e]	83.08 (76.63–89.52) [a, c]
UK	143	21.68 (14.92–28.43) [b, f]	143	20.98 (14.31–27.65) [d, e]	143	11.89 (6.58–17.19) [a]	92	6.52 (1.48–11.57) [a, e, f]	142	21.13 (14.41–27.84) [b, f, g]	89	15.56 (8.07–23.04) [a, e]	67.78 (58.12–77.43) [a, d, f]

*AMC* amoxicillin clavulanate, *3GC* third generation cephalosporins, *FLU* fluoroquinolones, *CN* gentamicin, *SXT* trimethoprim/sulfamethoxazole, *MDR* multidrug-resistant, *Full-S* fully-susceptible

^a^Countries: AT, Austria; BE, Belgium; DK, Denmark; FR, France; DE, Germany; EL, Greece; IT, Italy; NL, the Netherlands; PT, Portugal; RS, Serbia; ES, Spain; SE, Sweden; CH, Switzerland; UK, United Kingdom.

^b^95 % CI, 95 % Confidence interval

^c^Stat. Dif., Statistical significant differences. Countries with no statistical difference are marked with the same letter. Countries were compared by Fisher exact test with an alpha value of 0.05. Countries with less than ten tested isolates were not compared. Regarding MDR and FullS, only countries tested for all the considered antimicrobials were compared

*n*, Total number of *Escherichia coli* tested for the considered antimicrobial category

^d^MDR and FullS percentages do not include resistance to 3GC for Belgium and resistance to CN for the Netherlands

**Table 4:** Percentage of resistance in *Proteus* spp. by antimicrobial and country in 2012-2013

Country^a^	AMC		3GC		FLU		CN		SXT		Combined resistance		
	*n*	% R (95 % CI)^b^ [Stat. Dif.]^c^	*n*	% R (95 % CI)^b^ [Stat. Dif.]^c^	*n*	% R (95 % CI)^b^ [Stat. Dif.]^c^	*n*	% R (95 % CI)^b^ [Stat. Dif.]^c^	*n*	% R (95 % CI)^b^ [Stat. Dif.]^c^	*n*	% MDR (95 % CI)^b^ [Stat. Dif.]^c^	% FullS (95 % CI)^b^ [Stat. Dif.]^c^
AT	29	10.34 (0.00–21.43) [a, b, c]	29	0 [a, b]	29	17.24 (3.49–30.99) [a, b, c]	29	6.90 (0.00–16.12) [a, b]	29	37.93 (20.27–55.59) [a, b]	29	6.90 (0.00–16.12) [a]	55.17 (37.07–73.27) [a, b]
BE	143	2.10 (0.00–4.45) [a, g]	0	- -	135	28.15 (20.56–35.73) [a, d]	155	9.68 (5.02–14.33) [a]	154	35.06 (27.53–42.60) [a, b]	125^d^	4.80^d^ (1.05-8.55) -	57.60^d^ (48.94–66.26) -
DK	31	0 [a, d]	31	0 [a, b]	31	0 [e, f]	31	0 [a, b]	31	6.45 (0.00–15.10) [c]	31	0 [a, b]	93.55 (44.41–84.90) [c]
FR	215	7.44 (3.93–10.95) [b, d]	211	6.64 (3.28-9.99) [a]	212	17,92 (12.76-23.09) [b, g]	214	9.81 (5.83-13.80) [a]	216	27.78 (21.80-33.75) [a]	204	10.29 (6.12-14.46) [a]	66.67 (60.20-73.14) [a]
DE	10	0 [a, b, f]	10	10.00 (0.00–28.59) [a, b, c]	10	50.00 (19.01–80.99) [a, d]	10	0 [a, b, c]	10	20.00 (0.00–44.79) [a, c, d]	10	0 [a, b]	50.00 (19.01–80.99) [a, d]
EL	8	1R/7S -	0	- -	8	4R/4S -	0	- -	7	4R/3R -	0	- -	- -
IT	12	0 [a, b, c]	12	8.33 (0.00–23.97) [a, b, c]	12	41.67 (13.77–69.56) [a, d, g]	12	8.33 (0.00–23.97) [a, b, c]	12	66.67 (39.99–93.34) [b, e]	12	8.33 (0.00–23.97) [a, b, c]	16.67 (0.00–37.75) [d]
NL	261	6.13 (3.22–9.04) [a, b, e]	244	2.87 (0.77–4.96) [a]	260	8.85 (5.39–12.30) [c, e, h]	17	11.76 (0.00–27.08) [a, c]	260	27.31 (21.89–32.72) [a]	243^d^	3.29^d^ (1.05–5.54) -	69.96^d^ (64.19–75.72) -
PT	14	50.00 (23.81–76.19) [e]	15	33.33 (9.48–57.19) [c, d]	15	40.00 (15.21–64.79) [a, g, i]	15	33.33 (9.48–57.19) [c]	15	46.67 (21.42–71.91) [a, e]	14	42.86 (16.93–68.78) [c]	35.71 (10.61–60.81) [b, d, e]
RS	1	0R/1S -	1	1R/0S -	1	0R/1S -	1	0R/1S -	0	- -	0	- -	- -
ES	15	26.67 (4.29–49.05) [c, e, f]	13	15.38 (0.00–35.00) [a, d]	15	53.33 (28.09–78.58) [d, i]	9	2R/7S -	15	53.33 (28.09–78.58) [b, d, e]	7	2MDR -	4Full-S -
SE	170	2.35 (0.07–4.63) [a, f, g]	169	0 [b, e]	170	0.59 (0.00–1.74) [f]	170	0.59 (0.00–1.74) [b]	170	7.06 (3.21–10.91) [c]	169	0 [b]	91.12 (86.84–95.41) [c]
CH	17	0 [a, b, f]	17	0 [a, e]	17	23.53 (3.37–43.69) [a, g, h, I, j]	17	5.88 (0.00–17.07) [a, b, c]	17	35.29 (12.58–58.01) [a, e]	17	0 [a, b]	64.71 (41.99–87.42) [a, e]
UK	16	12.50 (0.00–28.70) [a, b, f]	16	0 [a, e]	16	0 [b, e, f, j]	11	0 [a, b, c]	15	33.33 (9.48–57.19) [a, e]	10	0 [a, b]	70.00 (41.60–98.40) [a, c, e]

*AMC* amoxicillin clavulanate, *3GC* third generation cephalosporins, *FLU* fluoroquinolones, *CN* gentamicin, *SXT* trimethoprim/sulfamethoxazole, *MDR* multidrug-resistant, *FullS* fully-susceptible

^a^Countries: AT, Austria; BE, Belgium; DK, Denmark; FR, France; DE, Germany; EL, Greece; IT, Italy; NL, the Netherlands; PT, Portugal; RS, Serbia; ES, Spain; SE, Sweden; CH, Switzerland; UK, United Kingdom.

^b^95 % CI, 95 % Confidence interval.

^c^Stat. Dif., Statistical significant differences. Countries with no statistical difference are marked with the same letter. Countries were compared by Fisher exact test with an alpha value of 0.05. Countries with less than ten tested isolates were not compared. Regarding MDR and FullS, only countries tested for all the considered antimicrobials were compared

*n*, Total number of *Proteus* spp. tested for the considered antimicrobial category

^d^MDR and FullS percentages do not include resistance to 3GC for Belgium and resistance to CN for the Netherlands

The lowest frequencies of AMC resistance in *E. coli* were detected in isolates from Denmark (2.88 %) and Belgium (4.29 %). *E. coli* from Portugal (48.15 %) had a significantly higher AMC resistance frequency (*P* < 0.05) when compared with all countries except for Spain, Italy and Greece (Table 3). Less than 15 % of *Proteus* spp. were resistant to AMC in all countries with exception of Portugal (50 %) and Spain (26.67 %) (Table 4).

*E. coli* resistance to 3GC had a similar distribution to what was seen for AMC (Fig. 2). The highest 3GC resistance frequencies were found in Southern countries, namely Portugal (31.25 %), Italy (24.64 %) and Spain (21.15 %) (Table 3). *Proteus* spp. 3GC resistance was lower than 5 % in Austria, Denmark, Sweden, Switzerland, United Kingdom and the Netherlands, whereas Portugal (33.33 %) and Spain (15.38 %) were the countries with the highest resistance levels (Table 4).

SXT resistance in Southern countries was higher than 25 and 45 % for *E. coli* and *Proteus* spp., respectively (Tables 3 and 4; Figs. 2 and 3). Sweden and Denmark had the lowest SXT resistance values (lower than 9 %). The remaining included countries had frequencies ranging between 10.21-21.13 % and 20–37.93 % in *E. coli* and *Proteus* spp., respectively (Tables 3 and 4).

*E. coli* FLU resistance was higher in the Southern countries and ranged from 29.03 % in Portugal to 31.88 % in Italy (Table 3). Concerning *Proteus* spp., Spain and Germany had around 50 % FLU resistance, followed by Italy and Portugal with around 40 % (Table 4). Sweden, Denmark, Belgium and the Netherlands had less than 10 % FLU resistant *E. coli*. Denmark, Sweden, United Kingdom and the Netherlands had less than 10 % FLU resistant *Proteus* spp. (Figs. 2 and 3).

Overall, *E. coli* CN resistance was lower than 16 % (Table 3). Regarding resistance to CN in *Proteus* spp. the same resistance frequency occurred with the exception of Portugal where a higher resistance frequency was recorded (33.33 %) (Table 4).

*E. coli* from Portugal, Spain and Italy, and *Proteus* spp. from Portugal showed the highest frequencies of MDR (Tables 3 and 4). As expected, Portugal was one of the countries with lowest FullS, both in *E. coli* (32.00 %) and *Proteus* spp. (35.71 %). Italy had even lower FullS in *Proteus* spp. (16.67 %). Most of the remaining countries had MDR levels lower than 10 %, with the exception of MDR *E. coli* from United Kingdom (15.56 %) and France (11 %). The highest *E. coli* and *Proteus* spp. FullS frequencies were found in Denmark and Sweden (Tables 3 and 4).

**Fig. 4:**
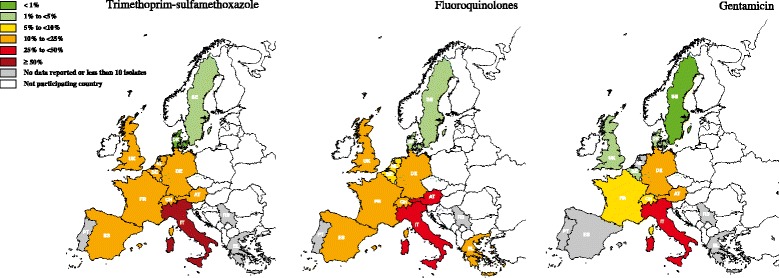
Percentage (%) of *Staphylococcus* spp. antimicrobial resistance by antimicrobial and country in the years 2012–2013. Countries: AT- Austria; BE- Belgium; DK- Denmark; FR- France; DE- Germany; EL- Greece; IT- Italy; NL- the Netherlands; PT- Portugal; RS- Serbia; ES- Spain; SE- Sweden; CH- Switzerland; UK- United Kingdom

Due to the limited number of *Staphylococcus* spp. isolates available, the percentage of resistance to antimicrobials was determined on fewer countries for this group of bacteria (Tables 5 and 6). In most countries, *Staphylococcus pseudintermedius* was the most frequently isolated followed by coagulase negative staphylococci (CoNS) (Table 5). In general, the overall antimicrobial resistance levels in Southern countries were higher than in Northern countries (Fig. 4) as seen in Gram-negative bacteria (Figs. 2 and 3).

**Table 5:** *Staphylococcus* spp. and methicillin-resistance by country in 2012–2013

	Staphylococci species by country						Methicillin-resistance within each group					
Country^a^	*N*	SA	SP	CoPS	CoNS	SPP	MRSA		MRSP		MRCoNS	
		% (95 % CI)^b^	% (95 % CI)^b^	% (95 % CI)^b^	% (95 % CI)^b^	% (95 % CI)^b^	*n* tested	R % (95 % CI)^b^	*n* tested	R % (95 % CI)^b^	*n* tested	R % (95 % CI)^b^
AT	78	7.69 (1.78–13.61)	19.23 (10.48–27.98)	6.41 (0.97–11.85)	56.41 (45.41–67.41)	10.26 (3.52–16.99)	5	1R/4S -	15	33.33 (9.48–57.19)	43	27.91 (14.50–41.31)
BE	122	14.75 (8.46–21.05)	52.46 (43.60–61.32)	0 -	32.79 (24.46–41.12)	0 -	18	5.56 (0–16.14)	64	12.50 (4.40–20.60)	40	25.0 (11.58–38.42)
DK	52	1.92 (0–5.66)	53.85 (40.30–67.40)	1.92 (0–5.66)	28.85 (16.53–41.16)	13.46 (4.18–22.74)	1	1S -	27	0 -	15	46.67 (21.42–71.91)
FR	242	8.26 (4.80–11.73)	72.73 (67.12–78.34)	0 -	19.01 (14.06–23.95)	0 -	20	40.0 (18.53–61.47)	0	- -	46	17.39 (6.44–28.34)
DE	64	4.69 (0–9.87)	50.0 (37.75–62.25)	0 -	39.06 (27.11–51.02)	6.25 (0.32–12.18)	0	- -	0	- -	0	- -
EL	10	0 -	10.0 (0–28.59)	0 -	0 -	90 (71.41–100)	-	- -	1	1R -	-	- -
IT	19	0 -	94.74 (84.70–100)	0 -	5.26 (0–15.30)	0 -	-	- -	18	50.0 (26.90–73.10)	0	- -
NL	365	4.93 (1.12–2.71)	47.95 (42.82–53.07)	0 -	47.12 (42.0–52.24)	0 -	0	- -	174	10.92 (6.29–15.55)	172	0.58 (0–1.72)
PT	7	1SA -	4SP -	0CoPS -	0CoNS -	2SPP -	1	1R -	4	2R/2S -	0	- -
RS	0	- -	- -	- -	- -	- -	-	- -	-	- -	-	- -
ES	13	0 -	0 -	15.38 (0–35.0)	46.15 (19.05–73.25)	38.46 (12.02–64.91)	-	- -	-	- -	0	- -
SE	325	8.62 (5.56–11.67)	53.54 (48.12–58.96)	2.46 (0.78–4.15)	32.31 (27.22–37.39)	3.08 (1.20–4.95)	28	0 -	174	1.15 (0–2.73)	105	4.76 (0.69–8.84)
CH	46	4.35 (0–10.24)	52.17 (37.74–66.61)	4.35 (0–10.24)	34.78 (21.02–48.55)	4.35 (0–10.24)	2	0R/2S -	20	10.00 (0–23.15)	15	66.67 (42.81–90.52)
UK	32	12.50 (1.04–23.96)	53.13 (35.84–70.41)	6.25 (0–14.64)	18.75 (5.23–32.27)	9.38 (0–19.47)	3	1R/2S -	12	8.33 (0–23.97)	3	1R/2S -

Staphylococci identification varied according to the country. Some countries identified staphylococci to species level, others to genus level and others included data on the coagulase test. Thus, the staphylococci results were grouped as follows: 1. *Staphylococcus aureus* (SA)*; 2. Staphylococcus pseudintermedius* (SP); 3. coagulase positive staphylococci (CoPS), 4. coagulase negative staphylococci (CoNS) and 5. other staphylococci (SPP). Group 2 includes staphylococci identified only as CoPS or staphylococci species known to be coagulase positive other than SA and SP. Group 3 includes staphylococci identified only as CoNS or staphylococci species known to be coagulase negative. Group 4 includes staphylococci identified as *Staphylococcus* spp

*MRSA* methicillin-resistant *Staphylococcus aureus, MRSP* methicillin-resistant *Staphylococcus pseudintermedius, MRCoNS* methicillin-resistant coagulase negative staphylococci

^a^Countries: AT, Austria; BE, Belgium; DK, Denmark; FR, France; DE, Germany; EL, Greece; IT, Italy; NL, the Netherlands; PT, Portugal; RS, Serbia; ES, Spain; SE, Sweden; CH, Switzerland; UK, United Kingdom

^b^95 % CI, 95 % Confidence interval

*N*, Total number of staphylococci

*n* tested, number of staphylococci tested for methicillin-resistance within each group

**Table 6:** Percentage of resistance in *Staphylococcus* spp. by antimicrobial and country in 2012–2013

Country^a^	FLU		CN		SXT	
	*n*	% R (95 % CI)^b^ [Stat. Dif.]^c^	*n*	% R (95 % CI)^b^ [Stat. Dif.]^c^	*n*	% R (95 % CI)^b^ [Stat. Dif.]^c^
AT	78	26.92 (17.08–36.77) [a, b]	78	19.23 (10.48–27.98) [a]	78	20.51 (11.55–29.47) [a, b]
BE	116	7.76 (2.89–12.63) [c, d]	107	3.74 (0.14–7.33) [b]	122	13.11 (7.12–19.10) [a, b, c]
DK	51	1.96 (0.00–5.77) [c, e, f]	51	3.92 (0.00–9.25) [b]	51	0 - [d]
FR	238	23.53 (18.14–28.92) [a, g]	237	5.06 (2.27–7.85) [b]	242	11.57 (7.54–15.60) [a, c]
DE	55	18.18 (7.99–28.38) [a, d, g]	55	10.91 (2.67–19.15) [a, b]	55	23.64 (12.41–34.86) [b]
EL	10	20.00 (0.00–44.79) [a, d, e,gN h]	0	- - -	9	1R/8S - -
IT	19	42.11 (19.90–64.31) [a]	19	26.32 (6.52–46.12) [a]	19	63.16 (41.47–84.85) [e]
NL	364	6.59 (4.04–9.14) [c, i]	9	1R/8S - -	365	11.51 (8.23–14.78) [c]
PT	6	3R/3S - -	7	2R/5S - -	7	1R/6S - -
RS	0	- - -	0	- - -	0	- - -
ES	13	15.38 (0.00-35.00) [a, d, e, h, i]	8	0R/8S - -	11	18.18 (0.00-40.97) [a, b, c]
SE	325	1.54 (0.20–2.88) [f]	325	0 - [c]	325	2.77 (0.99–4.55) [d]
CH	41	24.39 (11.25–37.54) [a, b]	41	9.76 (0.67–18.84) [a, b]	41	19.51 (7.38–31.64) [a, b, c]
UK	31	12.90 (1.10–24.70) [b, c, e, g]	22	4.55 (0.00–13.25) [a, b, c]	30	16.67 (3.33–30.00) [a, b, c]

*FLU* fluoroquinolones, *CN* gentamicin, *SXT* trimethoprim/sulfamethoxazole

^a^Countries: AT, Austria; BE, Belgium; DK, Denmark; FR, France; DE, Germany; EL, Greece; IT, Italy; NL, the Netherlands; PT, Portugal; RS, Serbia; ES, Spain; SE, Sweden; CH, Switzerland; UK, United Kingdom

^b^95 % CI, 95 % Confidence interval

^c^Stat. Dif., Statistical significant differences. Countries with no statistical difference are marked with the same letter. Countries were compared by fisher exact test with an alpha value of 0.05. Countries with less than ten tested isolates were not compared

*n*, Total number of *Staphylococcus* spp. tested for the considered antimicrobial category

Besides the limited number of staphylococci, methicillin-resistance results were also limited due to the identification only to genus level or lack of testing the appropriate antimicrobial surrogate. Denmark and Sweden showed the lowest *S. pseudintermedius* methicillin-resistance (MRSP) (0 and 1.15 %, respectively). The remaining countries had MRSP frequencies higher than 8 %, attaining 50 % in Italy. Methicillin-resistance was also high in CoNS (Table 5).

Staphylococci SXT resistance ranged from 2.77 to 63.16 % and showed similar geographical distribution to Gram-negative bacteria (Fig. 4). Among the participating countries, Staphylococci FLU resistance frequencies were higher in Italy (42.11 %) and again lower in Sweden (1.54 %) and Denmark (1.96 %), with the remaining countries varying between 6.59 and 26.92 % (Table 6). Italy, Austria, Germany and Switzerland CN resistant staphylococci frequencies ranged between 26.32 and 9.76 % while the remaining countries had less than 6 % (Table 6).

Regarding resistance temporal trends, most countries had no significant changes in *E. coli* resistance over the time periods considered (Table 7). Belgium showed a significant decrease in *E. coli* resistance to all antimicrobials and an increase in full susceptible isolates. Denmark (AMC, FLU, SXT), France (3GC, FLU), the Netherlands (3GC, FLU, SXT, MDR) and Sweden (CN, MDR) had also significant decreases in *E. coli* resistance over time (Table 7). However, the Netherlands (AMC) and Switzerland (CN) had a significant increase in *E. coli* resistance (Table 7). A rising trend was also detected in *Proteus* spp. FLU resistance from Belgium (Table 8).

**Table 7:** Temporal trends of antimicrobial resistance in *Escherichia coli* by country

Country^a^ (Years)	AMC	3GC	FLU	CN	SXT	MDR	FullS
	OR^b^ (95 % CI)^c^ *P* value	OR^b^ (95 % CI) ^c^ *P* value	OR^b^ (95 % CI) ^c^ *P* value	OR^b^ (95 % CI) ^c^ *P* value	OR^b^ (95 % CI) ^c^ *P* value	OR^b^ (95 % CI) ^c^ *P* value	OR^b^ (95 % CI) ^c^ *P* value
BE (2010–13)	**0.787** **(0.646**–**0.960)** **0.0180**	- - -	**0.749** **(0.635**–**0.882)** **0.0006**	**0.677** **(0.507**–**0.904)** **0.0081**	**0.796** **(0.695**–**0.912)** **0.0010**	**0.529** ^**d**^ **(0.393**–**0.712)** **<0.0001**	**1.275** ^**d**^ **(1.127**–**1.442)** **0.0001**
DK (2008–13)	**0.698** **(0.500**–**0.976)** **0.0357**	0.869 (0.646–1.169) 0.3533	**0.742** **(0.565**–**0.976)** **0.0325**	0.926 (0.620–1.384) 0.7086	**0.793** **(0.642**–**0.980)** **0.0316**	0.874 (0.615–1.242) 0.4528	**1.396** **(1.156**–**1.684)** **0.0005**
FR (2010–13)	0.885 (0.780–1.005) 0.0606	**0.859** **(0.749**–**0.987)** **0.0314**	**0.822** **(0.727**–**0.928)** **0.0016**	0.938 (0.734–1.200) 0.6121	0.960 (0.853–1.080) 0.4997	0.901 (0.782–1.037) 0.1448	**1.112** **(1.002**–**1.233)** **0.0456**
DE (2009–13)	1.029 (0.779–1.358) 0.8424	1.076 (0.805–1.438) 0.6211	1.185 (0.912–1.540) 0.2037	0.856 (0.520–1.409) 0.5397	1.040 (0.831–1.302) 0.7317	1.111 (0.801–1.541) 0.5295	0.941 (0.780–1.136) 0.5281
EL^e^ (2009–13)	1.534 (0.851–2.766) 0.1545	1.083 (0.586–2.003) 0.7992	0.924 (0.630–1.355) 0.6855	- - -	0.880 (0.596–1.301) 0.5229	- - -	- - -
IT (2009–2013)	1.175 (0.844–1.637 0.3391)	1.017 (0.749–1.383) 0.9127	0.828 (0.629–1.090) 0.1784	1.007 (0.700–1.449) 0.9686	0.769 (0.582–1.016) 0.0645	1.065 (0.761–1.490) 0.7147	1.248 (0.953–1.634) 0.1076
NL (2008–13)	**1.108** **(1.026**–**1.197)** **0.0088**	**0.465** **(0.402**–**0.539)** **<0.0001**	**0.916** **(0.841**–**0.999)** **0.0464**	0.682 (0.327–1.422) 0.3071	**0.917** **(0.859**–**0.978)** **0.0083**	**0.380** ^**d**^ **(0.320**–**0.450)** **<0.0001**	**1.648** ^**d**^ **(1494.**–**1.818)** **<0.0001**
PT (2008–13)	1.139 (0.913–1.419) 0.2482	1.187 (0.945–1.492) 0.1411	1.029 (0.823–1.287) 0.8029	1.222 (0.899–1.660) 0.2010	1.087 (0.867–1.364) 0.4680	1.156 (0.898–1.488) 0.2601	0.797 (0.629–1.010) 0.0608
ES (2010–13)	1.372 (0.855–2.201) 0.1899	1.551 (0.857–2.808) 0.1474	0.801 (0.529–1214) 0.2965	0.859 (0.467–1.578) 0.6238	0.752 (0.489–1.156) 0.1939	1.237 (0.677–2.258) 0.4897	0.944 (0.570–1.564) 0.8234
SE (2008–13)	0.976 (0.915–1.041) 0.4569	- - -	0.980 (0.827–1.147) 0.8018	**0.700** **(0.562**–**0.872)** **0.0015**	0.961 (0.892–1.037) 0.3059	**0.697** **(0.493**–**0.985)** **0.0407**	1.035 (0.965–1.110) 0.3341
CH (2008–13)	1.143 (0.905–1.445) 0.2621	1.116 (0.861–1.447) 0.4067	1.007 (0.841–1.205) 0.9426	**1.493** **(1.009**–**2.208)** **0.0451**	1.080 (0.901–1.294) 0.4050	1.189 (0.920–1.536) 0.1863	1.025 (0.877–1.197) 0.7594
UK (2008–13)	1.075 (0.857–1.357) 0.5194	1.106 (0.873–1.400) 0.4041	0.945 (0.739–1.208) 0.6511	1.306 (0.792–2.155) 0.2952	0.972 (0.797–1.185) 0.7778	1.355 (0.954–1.925) 0.0892	1.154 (0.950–1.401) 0.1492

*AMC* amoxicillin clavulanate, *3GC* third generation cephalosporins, *FLU* fluoroquinolones, *CN* gentamicin, *SXT* trimethoprim/sulfamethoxazole, *MDR* multidrug-resistant, *FullS* fully-susceptible

^a^BE, Belgium; DK, Denmark; FR, France; DE, Germany; EL, Greece; IT, Italy; NL, the Netherlands; PT, Portugal; ES, Spain; SE, Sweden; CH, Switzerland; UK, United Kingdom

^b^OR, Odds ratio

^c^95 % CI, 95 % Confidence interval

^d^MDR and FullS temporal trends do not include resistance to 3GC for Belgium and CN for the Netherlands

^e^Data regarding the years 2010 and 2012 were excluded from Greece resistance trends analysis since less than ten isolates were tested in those years

Statistically significant trends are highlighted in bold

**Table 8:** Temporal trends of antimicrobial resistance in *Proteus* spp. by country

Country^a^ (Years)	AMC	FLU	SXT
	OR^b^ (95 % CI)^c^ *P* value	OR^b^ (95 % CI) ^c^ *P* value	OR^b^ (95 % CI) ^c^ *P* value
BE (2010–13)	0.627 (0.312–1.259) 0.1889	**1.292 (1.006**–**1.659)** **0.0450**	0.891 (0.726–1.092) 0.2649
FR (2010–13)	0.945 (0.625–1.431) 0.7908	1.004 (0.764–1.319) 0.9770	0.954 (0.756–1.205) 0.6944
NL (2008–13)	1.092 (0.860–1.387) 0.4709	0.970 (0.829–1.135) 0.7067	1.010 (0.903–1.129) 0.8660
SE (2008–13)	0.892 (0.657–1.210) 0.4620	0.884 (0.494–1.583) 0.6788	0.941 (0.759–1.165) 0.5756

*AMC* amoxicillin clavulanate, *FLU* fluoroquinolones, *SXT* trimethoprim/sulfamethoxazole

Statistically significant trends are highlighted in bold

^a^BE, Belgium; DK, Denmark; FR, France; NL, the Netherlands; SE, Sweden; CH, Switzerland

^b^OR, Odds ratio

^c^95 % CI, 95 % Confidence interval

## Discussion

Published data on antimicrobial resistance in bacteria isolated from companion animal UTIs over Europe is scarce [19] and the comparison between studies is impaired by the use of different inclusion criteria and different time periods. Moreover, UTI resistance frequencies are usually reported together with susceptibility data from other sites of infection [11, 28], combining different bacteria genera [8, 9] and several countries [29, 30]. These facts impair the establishment of a global epidemiological overview of UTI bacteria resistance in Europe. This is the first large study to analyse antimicrobial susceptibility data of canine and feline isolates from several European countries allowing an epidemiological overview of UTI resistance trends in Europe.

In accordance to previous studies [7–9, 31, 32], *E. coli* was the most frequently isolated bacteria in dogs and cats. *Enterococcus* presented a significantly higher frequency in cats and *Proteus* spp. in dogs. While not compared in previous studies, this difference could be expected based on some published data focused on cats [7, 9] and dogs [32] separately.

One of the most important findings from this study was the overall higher resistance frequencies found in the Southern countries (Italy, Greece, Portugal and Spain) when compared with the Northern countries (Denmark and Sweden). The lower frequency of antimicrobial resistance in Northern countries, such as Sweden, is likely a consequence of the tight regulations and surveillance on antimicrobial prescribing and resistance in companion animals. In light of the present results, such strategies could be useful in aiming the reduction of antimicrobial resistance in the Southern countries.

### Resistance to Beta-Lactams

#### Amoxicillin-clavulanate

Considering that AMC is one of the most used antimicrobials in animals, the levels of resistance detected in this study are worrisome, especially in the Southern countries. Previous published reports showed different frequencies of AMC resistance in *E. coli* and in *Proteus* spp. that are likely due to the fact they report to different time frames and inclusion criteria [10, 32–37]. In the absence of clinical data it is not possible to know if this resistance relates to uncomplicated or complicated UTI [19]. Thus, these results need to be further investigated in order to establish whether AMC is a suitable empiric therapeutic choice for companion animals UTI in Southern Europe.

#### Third generation cephalosporins

Southern countries had also higher levels of resistance to 3GCs. Although Greece was not included due to limited data, considering that seven out of the nine tested isolates were resistant to 3GCs, one can expect the prevalence of 3GC resistance to be high. Previous studies in Portugal found a considerable lower 3GC resistance value (1.4 %) in *E. coli* from dogs in earlier years [10]. In the present work, the lower Swedish results for 3GC resistance in *E. coli* and *Proteus* spp. are in agreement with early studies [38]. Being of critical importance to humans [39], prudent use of 3GC is of upmost importance.

#### Methicillin-resistance

The frequency of methicillin-resistant staphylococci, especially *S. pseudintermedius* and CoNS, varied considerably between countries and confirmed previous reports on a low MRSP prevalence in Scandinavia compared to elsewhere in Europe [40]. Resistance to methicillin in coagulase-positive *Staphylococcus* (*S. aureus* and *S. pseudintermedius*) was detected in this study and is a great animal and public health concern [41]. Currently, the recommended methods for the detection of methicillin-resistance in *Staphylococci* are manly phenotypic but in some circumstances the molecular detection of the *mecA* gene is clinically and epidemiologically necessary [22, 23, 42]. This should be taken in consideration for the harmonization of veterinary susceptibility testing in Europe.

### Resistance to fluoroquinolones

In this study, high resistance frequencies towards the fluoroquinolones were found in *E. coli*, *Proteus* spp *and Staphylococcus* spp. isolates in the southern European countries but also in *Proteus* spp. from Germany, Belgium and Switzerland and *Staphylococcus* spp. from Austria, Switzerland and France. Several authors [32, 34, 43, 44] have reported lower FLU resistance frequencies than the ones found in this study, especially regarding the Southern countries [43, 44]. The results of high resistance frequencies towards the fluoroquinolones found in this study are concerning because fluoroquinolones are considered a good first choice for pyelonephritis treatment and should otherwise be used as a second line antimicrobial [19].

### Resistance to folate inhibitors and to aminoglicosides

In this study, resistance to SXT in Europe was high, especially in *E. coli* and *Proteus* spp.. The higher SXT resistance found in *Proteus* spp., than in *E. coli* from several European countries, is consistent with other reports [34, 36, 45]. Compared with previous studies, these results show a superior SXT resistance in *E. coli* and *Proteus* spp. from Italy and Portugal [10, 43] and *Staphylococcus* spp. from Belgium [36].

Also in agreement to previous studies, gentamicin was the antimicrobial with lower resistance in *E. coli*, *Proteus* spp. and *Staphylococcus* spp. all-over Europe [32, 34, 35, 37, 38, 43, 45]. Nevertheless, the distribution seemed to follow the same pattern, with increased resistance in Southern over Northern countries.

### Multidrug-resistance

Finally, MDR bacteria presented the worst scenario once again in *E. coli* from Southern countries and in *Proteus* spp. from Portugal. The emergence of MDR bacteria in companion animals has been previously described [46, 47] and represents a great therapeutic challenge and public health concern. However, MDR/FullS frequencies are seldom reported and published data account for different antimicrobials, thus impairing any comparisons with the present results [10, 32, 35, 38, 45].

### Trends in antimicrobial resistance

The surveillance of antimicrobial resistance is an important tool to guide the implementation of antimicrobial stewardship strategies. In this study, most countries had no significant changes in antimicrobial resistance over the time frame considered. Nevertheless, decreasing trends in antimicrobial resistance were found in *E. coli*. These encouraging trends were not detected in AMC and CN resistance in *E. coli* from the Netherlands and Switzerland, respectively, where an increasing trend was observed. Although no changes over time were detected in *E. coli* resistance against AMC and 3CGs in Portugal, the considerably lower resistance frequencies previously reported in earlier years [10], point to a possible increasing trend [33]. The same may be the case for *E. coli* AMC resistance in Germany and Switzerland [34, 35].

Despite reporting clear trends such as the difference in resistance between Northern and Southern countries, data from this study should be interpreted with caution. Due to the retrospective nature of this study, data on clinical history such as the type of UTI and previous antimicrobial treatment were unavailable. Furthermore, the use of laboratory data may represent a bias towards resistance, since urine cultures from complicated cases tend to be requested more often than simple uncomplicated UTI [8, 31]. These limitations are not restricted to certain countries, and are therefore not likely to hamper comparison of data across borders. Given the limitations of retrospective studies, a veterinary European surveillance network gathering data prospectively on antimicrobial resistance, as well as, on clinical data is of the upmost importance to facilitate development of national evidence-based guidelines that take into consideration type of UTI, local regulations and patterns of antimicrobial resistance.

The use of different susceptibility testing methods and different clinical breakpoints is considered a major limitation. The lack of harmonization became evident in this study when trying to compare 3GC and methicillin-resistance. Although it also happens in the well-established EARS network reports of resistance on bacteria from human invasive infections [18], this limitation weakens the comparison of resistance between countries in the present and future surveillance studies. This harmonization would allow future within and between countries resistance frequencies comparisons over time and would also provide relevant information on the impact of different antimicrobial usage policies. Thus, the authors agree that the harmonization of methods and interpretative criteria in veterinary medicine should be a priority. The role of the new veterinary committee on antimicrobial susceptibility testing VetCAST [48] may be crucial in this harmonization process. Despite these limitations, the results from this study provide relevant and updated information on the current antimicrobial resistance in UTI bacteria from companion animals in Europe. Similar studies should also be conducted regarding other types of infection to improve the awareness on the European distribution of antimicrobial resistance in companion animals. Ideally, monitoring of companion animal antimicrobial resistance should be implemented in Europe, as it is the case for food producing animals. Such surveillance would provide crucial information to promote the appropriate use of antimicrobial and therefore limit the spread of resistance.

## Conclusions

This work brings new insights into the current scenario of the European antimicrobial resistance bacteria isolated from companion animals with UTI. An important finding from this study was the higher frequency of resistance in Southern European countries (Italy, Greece, Spain, Portugal) when compared to Northern European countries (Denmark, Sweden). Furthermore, there is an evident need to harmonize methods and interpretative criteria in veterinary medicine. Given the limitations of retrospective studies, an European surveillance network gathering data on antimicrobial resistance is of the upmost importance to facilitate the development of national evidence-based guidelines.

## Abbreviations

3GC: Third generation cephalosporins
95 % CI: 95 % confidence interval
AMC: Amoxicillin-clavulanate
AMP: Ampicillin
AT: Austria
BE: Belgium
CAZ: Ceftazidime
CH: Switzerland
CIP: Ciprofloxacin
CLSI: Clinical and Laboratory Standards Institute
CN: Gentamicin
CoNS: Coagulase negative staphylococci
CoPS: Coagulase positive staphylococci
CPD: Cefpodoxime
CTX: Cefotaxime
CVN: Cefovecin
DE: Germany
DK: Denmark
EARS-Net: European Antimicrobial Resistance Surveillance Network
EFT: Ceftiofur
EL: Greece
ENR: Enrofloxacin
ES: Spain
FLU: Fluoroquinolones
FOX: Cefoxitin
FR: France
FullS: Full-susceptibility
IT: Italy
MAR: Marbofloxacin
MDR: Multidrug-resistant
MRCoNS: Methicillin-resistant coagulase negative staphylococci
MRSA: Methicillin-resistant *Staphylococcus aureus*
MRSP: Methicillin-resistant *Staphylococcus pseudintermedius*
NL: the Netherlands
OR: Odds ratio
OX: Oxacillin
P: Penicillin
PT: Portugal
R: Full resistant
RS: Serbia
S: Susceptible
SA: *Staphylococcus aureus*
SE: Sweden
SP: *Staphylococcus pseudintermedius*
Stat. Dif.: Statistical significant differences
SXT: Trimethoprim/sulfamethoxazole
UK: United Kingdom
UTI: Urinary tract infection
